# Impact of Metabolic Surgery on Obesity Outcomes and Framingham Risk Score in Iranian Diabetic Patients: A Short- and Long-Term Follow-Up Study

**DOI:** 10.7759/cureus.91245

**Published:** 2025-08-29

**Authors:** Ferdos Zaman, Zahra Farhangiyan, Maryam Alamdari, Leila Moradi, Amir Ashrafi

**Affiliations:** 1 Endocrinology, Diabetes Research Center, Health Research Institute, Ahvaz Jundishapur University of Medical Sciences, Ahvaz, IRN; 2 General Surgery, School of Medicine, Ahvaz Jundishapur University of Medical Sciences, Ahvaz, IRN

**Keywords:** bariatric surgery, cardiovascular risk factor, diabetes mellitus, framingham risk score, obesity outcomes

## Abstract

Type 2 diabetes mellitus (T2DM) and obesity are closely interlinked conditions, contributing significantly to health complications. Metabolic surgery (MS) could help to improve metabolic conditions, including diabetes, hypertension, and hyperlipidemia, which are significant risk factors for cardiovascular disease. This study aimed to evaluate the short-term and long-term effects of MS on obesity outcomes and the Framingham Risk Score (FRS) in Iranian diabetic patients. This multicenter, retrospective study included T2DM patients with body mass index (BMI) ≥ 30 kg/m² who underwent MS in Ahvaz, Iran, from 2019 to 2023. Of the 151 patients, 105 with a follow-up of at least one year were included. Among them, 29 patients (27.6%) had a long-term follow-up (four to five years). Anthropometric and biochemical parameters, comorbidities, and FRS were evaluated at baseline and at one-year and long-term follow-ups. One year postoperatively, there were significant reductions in weight, BMI, systolic and diastolic blood pressure, FRS, triglycerides, low-density lipoprotein (LDL), liver enzymes, fasting blood glucose, and hemoglobin A1c (HbA1c), with an increase in high-density lipoprotein (HDL) levels. The percentage of total weight loss (%TWL) and excess weight loss (%EWL) were 29.13 ± 8.42 and 71.03 ± 20.88, respectively. The median FRS decreased from 11.70 pre-surgery to 4.50 and 6.30 at one-year and long-term follow-ups (P<0.0001). Complete remission of T2DM, hypertension, and hyperlipidemia was achieved in 75.2%, 48.2%, and 35.1% of patients, respectively, and improvements were sustained throughout the follow-up period. The type of MS had no significant impact on changes in weight or BMI (P>0.05). Surgery-related side effects were observed in four cases (3.80%). MS is a safe and effective procedure for reducing obesity-related complications, including T2DM, hypertension, hyperlipidemia, and cardiovascular risk in both the short and long term. These findings highlight the promising potential of MS in managing diabetes.

## Introduction

Obesity is a growing global health concern, affecting 13% of adults worldwide [1]. Obesity has a profound impact on multiple body systems and is associated with increased risk of chronic and metabolic disorders, including type 2 diabetes mellitus (T2DM), hypertension, dyslipidemia, cardiovascular diseases (CVDs), stroke, and premature death [2-4]. Furthermore, obesity-related mortality is primarily attributed to diabetes and cardiovascular causes [3]. The global increase in both obesity and T2DM is alarming. A significant proportion of obese individuals develop diabetes, and obesity is the strongest risk factor for T2DM [5]. On the other hand, nearly 90% of individuals with T2DM are either overweight or obese, indicating a strong association between the two conditions and highlighting the need for effective and sustainable treatments [4,6].

Various strategies have been implemented for the long-term management of obesity, including lifestyle modification, dietary changes, pharmacological treatments, and surgical interventions [6]. Among these, surgical procedures, commonly referred to as bariatric surgery, are the most effective and sustainable treatment for severe obesity and T2DM. Today, they are also known as metabolic surgery (MS) due to their effects not only on weight loss but also on metabolic complications associated with obesity [7,8]. The American Diabetes Association (ADA) recommends MS as a treatment option for weight loss and glycemic control in diabetic individuals with a body mass index (BMI) ≥ 30 kg/m² (or ≥ 27.5 kg/m² in Asian Americans) [9]. Metabolic surgeries induce weight loss through restrictive and malabsorptive mechanisms. Restrictive procedures, such as sleeve gastrectomy (SG), limit food intake by reducing stomach size, while malabsorptive procedures, such as Roux-en-Y gastric bypass (RYGB), reduce nutrient absorption by altering the length of the digestive tract [10].

CVDs remain the leading cause of mortality globally, and obesity is a well-established risk factor for CVD [11]. However, limited studies have assessed the impact of MS on cardiovascular risk, particularly using the Framingham Risk Score (FRS), which predicts the 10-year risk of cardiovascular events [12]. Despite the well-established benefits of MS in improving obesity-related complications, international findings may not be generalizable to Iranian populations due to ethnic and racial variations. Considering the increasing prevalence of obesity and its complications, especially among diabetic patients, a comprehensive evaluation of MS outcomes across different regions is essential. Therefore, the present study aimed to assess the effects of common MSs on obesity outcomes and the FRS in Iranian obese patients with T2DM.

## Materials and methods

Study design

This retrospective multicenter study was conducted among diabetic patients with a BMI of ≥30 kg/m² who underwent MS at Golestan, Naft, and Mehr Hospitals in Ahvaz, Iran, from 2019 to 2023. The study protocol was approved by the Research Ethics Committee of Golestan Hospital of Ahvaz Jundishapur University of Medical Sciences, Ahvaz, Iran (ethical code: IR.AJUMS.HGOLESTAN.REC.1403.116). This study was conducted in accordance with the guidelines established by the Declaration of Helsinki.

Patients

The sample size was calculated using G*Power software (Heinrich Heine University Düsseldorf, Düsseldorf, Germany) and the two-tailed test, with a 95% confidence interval (α=0.05), 90% power (β=0.1), and a 0.32 effect size (considering alanine aminotransferase (ALT) changes after MS in diabetic patients in the study by Alamro et al. [2]) to be at least 103 patients, and assuming a 5% dropout rate, 109 patients should be included in the study. Participants were selected using a convenience sampling method based on the inclusion and exclusion criteria.

Inclusion Criteria

Patients with T2DM according to the ADA guidelines [13], aged between 18 and 65 years, undergoing primary MS including laparoscopic sleeve gastrectomy (LSG), RYGB, and mini-gastric bypass (MGB), with a BMI ≥ 30 kg/m^2^ with or without obesity-related comorbidities, and who completed one year of follow-up visits were included in the study.

Exclusion Criteria

Patients with active substance use, history of organ transplant, history of cancer, history of severe liver disease with ascites, pregnancy during follow-up, less than one year of follow-up, or incomplete clinical data after surgery were excluded from the study.

**Figure 1:**
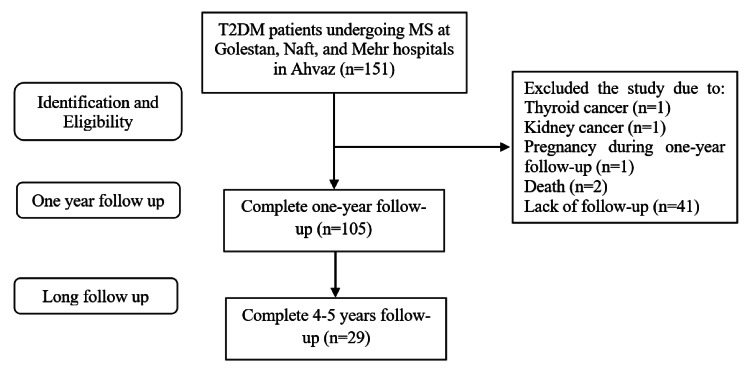
Flowchart of the study T2DM: type 2 diabetes mellitus; MS: metabolic surgery

Of the 151 patients reviewed, 105 who had a follow-up of at least one year were included in this study, and among them, 29 patients (27.6%) had long-term follow-up (four to five years) (Figure 1). Patients with incomplete data were excluded from the study to maintain data quality and reliability. Only patients with fully available records were included in the final analysis.

Data collection

The patients' data were collected retrospectively from the patients' electronic medical records in the hospital database. This information included demographic characteristics, anthropometric measurements, medical history (comorbidities and medications), smoking status, blood pressure (BP), and biochemical parameters. All data were collected at baseline (before MS), one year (short-term follow-up), and four to five years after MS (long-term follow-up). Surgery-related adverse events, including gastrointestinal bleeding, anemia, and mortality, were also assessed and recorded. Laboratory parameters that were assessed included hemoglobin (Hb), mean corpuscular volume (MCV), fasting blood glucose (FBS), hemoglobin A1C (HbA1c), HDL-cholesterol (HDL-C), LDL-cholesterol (LDL-C), triglycerides (TG), total cholesterol (TC), ALT, aspartate aminotransferase (AST), alkaline phosphatase (ALP), and serum vitamin D (25-OH Vitamin D). For all tests, sampling was performed after an eight-hour fast.

Hypertension was defined as having a systolic blood pressure (SBP) of ≥140 mmHg, a diastolic blood pressure (DBP) of ≥90 mmHg, or the use of antihypertensive medications [14]. Hyperlipidemia was defined as the presence of either TC ≥ 200 mg/dL, TG ≥ 150 mg/dL, LDL-C ≥ 130 mg/dL, and HDL-C < 40 mg/dL or using lipid-lowering drugs [15]. FRS for CVD risk was evaluated using the Medscape online calculator (available at https://reference.medscape.com/calculator/252/framingham-risk-score-2008). The main components for estimated FRS were gender, age, smoking status, TC, HDL, SBP, use of medication for hypertension, diabetes, and known vascular disease. The type of MS was selected for each patient according to standard protocols and indications, and all procedures were performed using standardized techniques. All patients received nutritional advice for obesity and underlying diseases, as well as physical activity counseling after surgery. In addition, all patients received the necessary treatment for DM and other comorbidities following surgery.

Outcome definitions

The aim of this study was to determine the weight loss and comorbidity remission following MS. Postoperative weight loss was expressed in terms of percentage of total weight loss (%TWL), percentage of excess weight loss (%EWL), and percentage of BMI loss (%BMIL) [16]. The following equations were used: BMI = weight (kg)/height^2^ (m); %BMIL = (Initial BMI - post-op BMI)/(Initial BMI) × 100; %TWL = (Initial weight - post-op weight)/(Initial weight) × 100; %EWL = (Initial weight - post-op weight)/(Initial weight - ideal body weight) × 100. The ideal weight was defined as the weight corresponding to a BMI of 25 kg/m² [16].

The status of comorbidities, including T2DM, hypertension, dyslipidemia, and FRS, was evaluated based on medical history, medications, and laboratory tests during preoperative, one-year, and four- to five-year postoperative visits. This classification was based on standards reported by the American Society for Metabolic and Bariatric Surgery (ASMBS) into four categories: remission (complete resolution of the comorbidity), improvement (partial remission), unchanged, and recurrence (worsening of the condition after remission) [16].

Statistical analysis

Continuous variables were reported as mean ± standard deviation (SD) or median (Q1-Q3), and categorical variables were expressed as number (percentage). The normality of data distribution was assessed using the Kolmogorov-Smirnov test. Independent t-tests (Mann-Whitney U tests) and ANOVA were used to compare quantitative variables, while chi-square tests were applied to compare qualitative variables. Spearman's correlation coefficient was used to assess associations between continuous variables. For intragroup comparisons over time, one-way ANOVA with repeated measures (or the Friedman test) was used. Comparisons between two time points were performed using a paired t-test (or Wilcoxon signed-rank test). McNemar's test was used to evaluate changes in comorbidity status after surgery. In some analyses, patients who underwent RYGB and MGB procedures were combined into one group, labeled as gastric bypass (GB), due to the small number of subjects. All statistical analyses were conducted using IBM SPSS Statistics for Windows, Version 22 (Released 2013; IBM Corp., Armonk, New York), and a P-value < 0.05 was considered statistically significant.

## Results

The study included a total of 105 patients, with a mean age of 40.37 ± 9.40 years (range, 20-65 years) (Table 1). The long-term follow-up rate (four to five years) was 27.6% (n = 29). The type of MS was LSG in 52 patients (49.5%), MGB in 31 (29.5%), and RYGB in 22 patients (21.0%).

**Table 1:** Preoperative characteristics of patients Data are shown as mean ± SD for continuous variables and number (%) for categorical variables. MS: metabolic surgery; LSG: laparoscopic sleeve gastrectomy; RYGB: Roux-en-Y gastric bypass; MGB: mini-gastric bypass; BMI: body mass index.

Characteristic		Values
Age (year)		40.37 ± 9.40
Gender	Female	77 (73.3)
	Male	28 (26.7)
Active smoker		2 (1.9)
Type of MS	LSG	52 (49.5)
	MGB	31 (29.5)
	RYGB	22 (21.0)
Hypertension		56 (53.3)
Hyperlipidemia		57 (54.3)
Atrial fibrillation		1 (0.95)
Coronary artery disease		4 (3.8)
Height (cm)		163.99 ± 9.59
Weight (kg)		116.02 ± 17.59
BMI (kg/m^2^)		42.81 ± 4.15
Medication for diabetes	Oral	59 (56.2)
	Insulin	4 (3.8)
	Oral + insulin	12 (11.4)
	No medication	30 (28.6)

**Figure 2:**
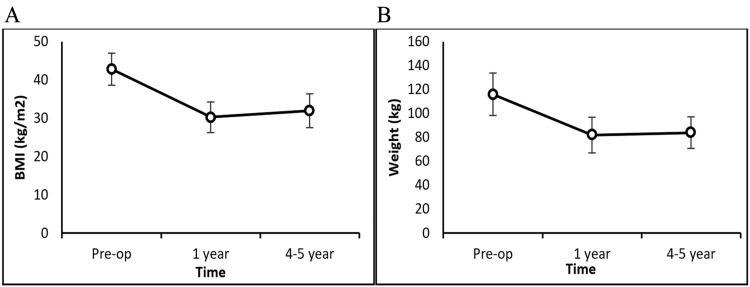
Weight and BMI change pre- and postoperatively A: Mean BMI (kg/m^2^) at baseline, one year, and four to five years after surgery B: Mean body weight (kg) at baseline, one year, and four to five years after surgery BMI: body mass index

Mean ± SD weight decreased significantly from 116.02 ± 17.59 kg preoperatively to 82.02 ± 14.81 kg at the one-year follow-up (P<0.0001). Similarly, the mean ± SD BMI decreased from 42.81 ± 4.15 kg/m² preoperatively to 30.27 ± 4.00 kg/m² at the one-year follow-up (P<0.0001) (Figure 2, Table 2). At the one-year follow-up, the mean ± SD %TWL, %EWL, and %BMIL were 29.13 ± 8.42, 71.03 ± 20.88, and 29.11 ± 8.33, respectively. Repeated-measures ANOVA showed that changes in weight and BMI were statistically significant over time (P<0.0001). The paired t-test indicated that the mean weight and BMI at one-year and four- to five-year follow-ups were significantly lower than baseline values (P<0.0001) (Table 2). However, no significant change was observed between the patients' weight and BMI during the one-year and long-term follow-up periods (P=0.345 and P=0.249). Similarly, %TWL, %EWL, and %BMIL were not significantly different at one year compared to four- to five-year assessments (P>0.05).

**Table 2:** Weight and BMI changes over time * P-values ​​were determined by one-way ANOVA with repeated measures or paired t-tests. ^#^ Indicates a significant change (P<0.05) compared to preoperative values. Data are shown as mean ± SD. BMI: body mass index; TBWL: total body weight loss; %TWL: percent total weight loss; %EWL: percent excess weight loss; %BMIL: percent BMI loss; N/A: not applicable.

Variable	Preoperative	Postoperative 1 year	Postoperative 4-5 years	P-value*
Weight (kg)	116.02 ± 17.59	82.02 ± 14.81^#^	83.97 ± 13.17^#^	<0.0001
BMI (kg/m^2^)	42.81 ± 4.15	30.27 ± 4.00^#^	31.96 ± 4.41^#^	<0.0001
TBWL (kg)	N/A	34.00 ± 11.55	30.59 ± 9.47	0.345
%TWL	N/A	29.13 ± 8.42	26.64 ± 7.28	0.352
%EWL	N/A	71.03 ± 20.88	63.92 ± 19.96	0.483
BMI change (kg/m^2^)	N/A	12.55 ± 4.14	11.48 ± 2.95	0.249
%BMIL	N/A	29.11 ± 8.33	26.57 ± 7.21	0.321

At the one-year follow-up, 13 patients (12.38%) achieved their ideal weight. Among the 29 patients with the long follow-up, two patients (6.90%) had achieved their ideal weight. Moreover, in the long-term follow-up after MS, 20 patients (68.97%) maintained their initial weight loss.

A significant diﬀerence was found between the preoperative and postoperative values of mean SBP and DBP, FBS, HbA1c, TC, HDL-C, LDL-C, TG, and liver function tests (ALT, AST, ALP) at one-year and four- to five-year follow-ups (Table 3). However, no statistically significant difference was observed between the one-year and four- to five-year follow-ups (P>0.05). The mean ± SD FBS decreased from 157.22 ± 54.83 mg/dL at baseline to 99.91 ± 15.47 mg/dL at one-year follow-up (P<0.0001). Similarly, the mean ± SD HbA1c decreased from 7.28 ± 1.47% to 5.46 ± 0.6% (P<0.0001).

**Table 3:** Postoperative changes in cardio-metabolic and biochemical variables * P-values ​​were determined by one-way ANOVA with repeated measures or Friedman's test. ^#^ Indicates a significant change (P<0.05) compared to preoperative values. Data are shown as mean ± SD for variables with normal distribution and as median (Q1-Q3) for variables with skewed distribution. SBP: systolic blood pressure; DBP: diastolic blood pressure; FRS: Framingham Risk Score; FBS: fasting blood sugar; HbA1c: hemoglobin A1C; HDL-C: high-density lipoprotein cholesterol; LDL-C: low-density lipoprotein cholesterol; TG: triglyceride; TC: total cholesterol; AST: aspartate aminotransferase; ALT: alanine aminotransferase; ALP: alkaline phosphatase; Hb: hemoglobin; MCV: mean corpuscular volume; 25-OH Vit D: 25-hydroxy vitamin D.

Variable	Preoperative	Postoperative 1 year	Postoperative 4-5 years	P-value*
SBP (mmHg)	127.00 ± 10.50	117.57 ± 7.31^#^	118.28 ± 5.87^#^	<0.0001
DBP (mmHg)	78.48 ± 7.41	75.62 ± 6.15^#^	76.90 ± 5.41	<0.0001
FRS (%)	11.70 (6.30–18.50)	4.50 (2.80–7.90)^#^	6.30 (3.45–9.85)^#^	<0.0001
FBS (mg/dL)	137 (126–169)	96 (91–104)^#^	100 (92–104)^#^	<0.0001
HbA1C (%)	6.80 (6.30–7.85)	5.30 (5.00–5.60)^#^	5.40 (5.00–5.50)^#^	<0.0001
HDL-C (mg/dL)	45.16 ± 9.52	52.08 ± 9.36^#^	54.97 ± 10.15^#^	<0.0001
LDL-C (mg/dL)	116.99 ± 36.71	101.26 ± 32.24^#^	108.41 ± 34.43	0.024
TG (mg/dL)	163.23 ± 63.02	109.57 ± 36.26^#^	114.69 ± 51.24^#^	<0.0001
TC (mg/dL)	195.63 ± 46.46	176.71 ± 36.73^#^	186.62 ± 37.97	0.014
AST (U/L)	24 (18–34)	20 (16.5–25)^#^	17 (15–25.5)^#^	<0.0001
ALT (IU/L)	27 (19–42.5)	18 (14.5–24.5)^#^	16 (12.5–26)^#^	<0.0001
ALP (IU/L)	190 (153–218)	182 (150–220)^#^	198 (162–222.5)	<0.0001
Hb (g/dL)	12.96 ± 1.42	12.72 ± 1.38	12.40 ± 1.62	0.233
MCV (fL)	83.0 (79.5–88.0)	85.0 (80.0–87.0)	85.0 (79.0–87.0)	0.611
25-OH Vit D (ng/mL)	28.0 (19.0–32.0)	30 (25.0–40.0)^#^	25 (20.7–27.0)	0.033

**Figure 3:**
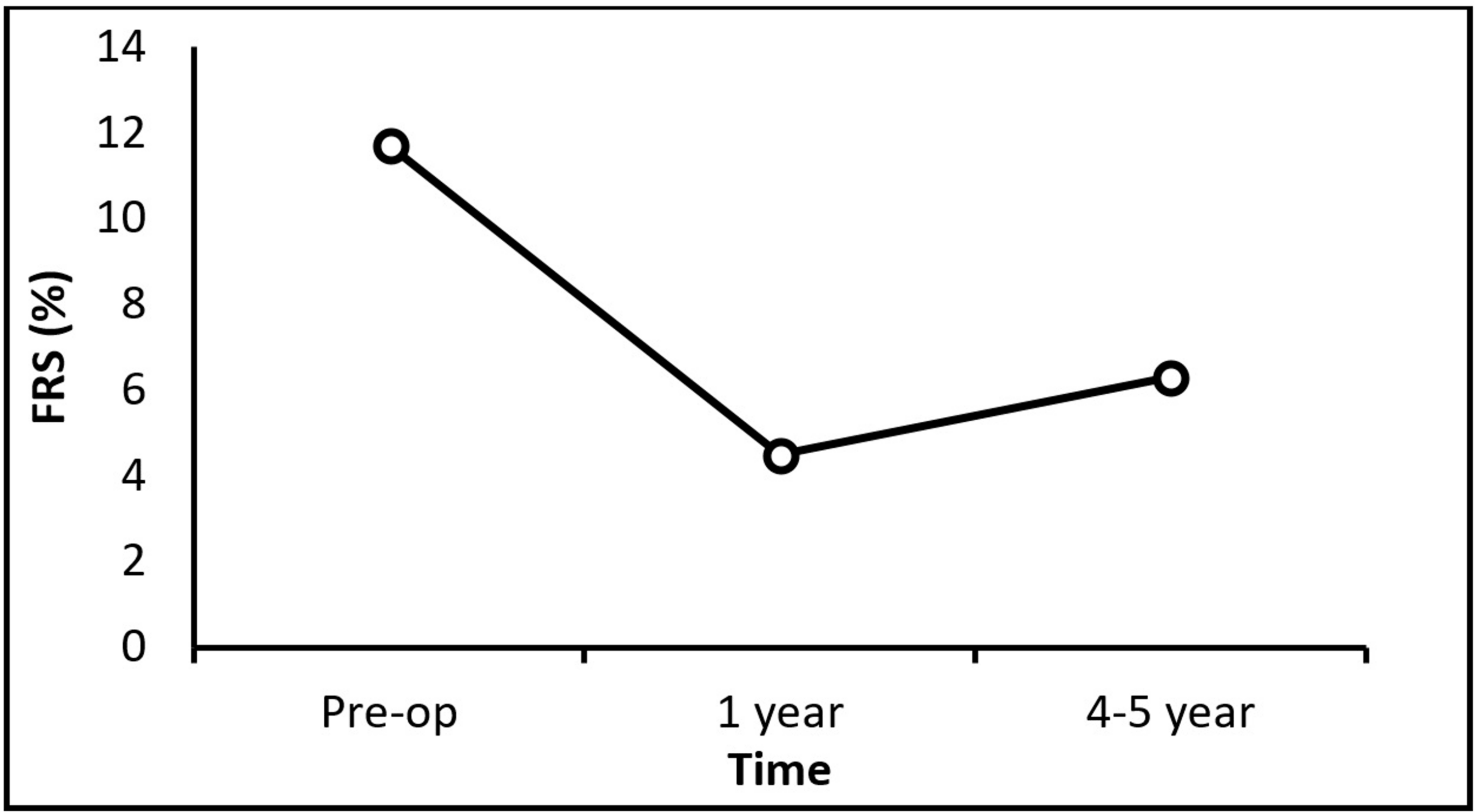
Framingham Risk Score (FRS) change pre- and postoperatively

Furthermore, changes in FRS over time were statistically significant (P<0.0001). Pairwise comparisons revealed that postoperative FRS values at both the one-year and four- to five-year follow-ups were significantly lower than preoperative values. However, there was no significant difference between the one-year and four- to five-year follow-up (P=0.218) (Figure 3; Table 3). In contrast, no significant changes were observed in Hb and MCV across the time points (P=0.233 and P=0.611, respectively). On the other hand, a significant change was observed in the serum 25-OH vitamin D level after a one-year follow-up (P=0.001) but not after four to five years (P=0.446) (Table 3).

Remission of T2DM was achieved in 79 patients (75.2%), and improvement was seen in 26 patients (24.8%) at the one-year follow-up. In the long-term follow-up, remission was observed in 20 out of 29 patients (69.0%), improvement in six (20.7%), and one patient (3.4%) remained unchanged. Two patients (6.9%) experienced recurrence after complete remission (Table 4). Furthermore, insulin use decreased from 16 patients (15.23%) before surgery to two patients (1.90%) at the one-year follow-up. Complete remission of hypertension and hyperlipidemia was observed in 48.2% and 35.1% of patients, respectively, at the one-year follow-up, and this improvement remained stable throughout the long-term follow-up. McNemar's test results demonstrated a significant reduction in the frequency of T2DM, hypertension, and hyperlipidemia at one-year and long-term follow-ups (P<0.0001 for all conditions). However, no significant difference was found between the one-year and long-term follow-ups (P>0.05) (Table 4).

**Table 4:** Status of comorbidities pre- and postoperatively * P-values ​​were determined using McNemar's test.

Comorbidities	Preoperative n(%)	Postoperative 1 year			Postoperative 4-5 years				P-value*
		Remission	Improvement	Unchanged	Remission	Improvement	Unchanged	Recurrence	
Diabetes mellitus	105 (100)	79 (75.2)	26 (24.8)	0	20 (69.0)	6 (20.7)	1 (3.4)	2 (6.9)	<0.0001
Hypertension	56 (53.3)	27 (48.2)	26 (47.3)	3 (5.5)	5 (27.8)	13 (72.2)	0	0	<0.0001
Hyperlipidemia	57 (54.3)	20 (35.1)	31 (54.4)	6 (10.5)	4 (28.6)	5 (35.7)	1 (7.1)	4 (28.6)	<0.0001

There was no significant difference in the frequency of T2DM and hyperlipidemia between different MS procedures at the one-year (P=0.193 and P=0.449, respectively) and long-term follow-ups (P=0.322 and P=0.085, respectively). However, the frequency of hypertension at one-year (P=0.032) and long-term (P=0.017) follow-up was significantly higher in patients undergoing LSG than in the GB (RYGB+MGB). Moreover, no new cases or worsening preexisting CVD (including myocardial infarction, coronary artery disease, stroke, atrial fibrillation, or heart failure) were observed during the one-year follow-up. Surgery-related adverse events were observed in four patients (3.80%), including three cases of cholecystectomy and one case of diabetic ketoacidosis.

The mean weight reduction was significantly greater in men than in women one year after surgery (P=0.017) (Table 5). However, the BMI changes at one-year and long-term follow-up (four to five years) after MS did not significantly differ by gender (P=0.855 and P=0.610, respectively). There were no significant differences in weight and BMI changes at one-year and long-term follow-up after MS based on the presence of hypertension and hyperlipidemia or the type of MS (P>0.05) (Table 5). When comparing two surgical groups (LSG vs. GB), the mean weight loss was greater in the GB group than in the LSG group at the one-year follow-up (36.27±12.07 vs. 31.69±10.61; P=0.041). However, no significant differences were observed in long-term BMI and weight changes (P<0.05).

**Table 5:** Weight changes according to variables * P-values ​​were determined by an independent t-test or ANOVA. Weight changes (kg) are presented as the mean ± standard deviation, compared to preoperative values. LSG: laparoscopic sleeve gastrectomy; MGB: mini-gastric bypass; RYGB: Roux-en-Y gastric bypass.

Variable		Weight changes after one year	P-value*	Weight changes after 4-5 years	P-value*
Gender	Male	38.44 ± 11.09	0.017	34.82 ± 11.11	0.226
	Female	32.38 ± 11.35		29.48 ± 8.94	
Hypertension	No	34.34 ± 11.45	0.780	33.56 ± 9.75	0.282
	Yes	33.70 ± 11.73		29.25 ± 9.28	
Hyperlipidemia	No	35.13 ± 11.77	0.385	30.21 ± 11.13	0.829
	Yes	33.04 ± 11.38		30.99 ± 7.70	
Metabolic surgery, n(%)	LSG	31.69 ± 10.61	0.106	28.17 ± 9.17	0.161
	MGB	35.51 ± 10.54		35.70 ± 10.13	
	RYGB	37.34 ± 14.32		34.00 ± 5.19	

No significant correlation was found between changes in weight, BMI, lipid profile, and BP (SBP and DBP) at one-year and long-term follow-up and changes in FBS and HbA1C in one-year follow-up and the patients' age (P>0.05). However, a significant and positive correlation was observed between the FRS changes one year after MS and patients' age (r=0.253, P=0.011), indicating that older patients experienced greater improvements in FRS. Furthermore, long-term changes in FBS and HbA1c after surgery were significantly correlated with the patients' age (r=0.406, P=0.029 and r=0.565, P=0.001, respectively); that is, older patients exhibited greater reductions in these parameters. The mean FRS change one year after MS was greater in men (P=0.001) and in patients with hypertension (P<0.0001) and hyperlipidemia (P=0.033) (Table 6).

**Table 6:** FRS changes according to patient characteristics * P-values ​​were determined by the Mann-Whitney U test. FRS: Framingham Risk Score

Variable		FRS changes after one year	P-value*
Gender	Male	10.34 ± 6.05	0.001
	Female	6.21 ± 5.61	
Hypertension	No	4.43 ± 3.27	<0.0001
	Yes	9.65 ± 6.68	
Hyperlipidemia	No	5.92 ± 5.38	0.033
	Yes	8.32 ± 6.25	

## Discussion

The results of this study demonstrated that MS in patients with T2DM and BMI ≥ 30 kg/m² resulted in a significant and sustained reduction in weight (both TBWL and EWL) and BMI during the one-year and long-term (four to five years) follow-ups. In the long-term follow-up, 68.97% of patients maintained their primary weight loss, suggesting that MS in diabetic patients without considering underlying diseases results in significant and sustainable weight loss. These results are consistent with previous studies by Alamro et al. [2], Moradi et al. [17], Vennapusa et al. [18], and Park et al. [19], which reported that common metabolic surgical procedures such as LSG and GB have substantial effects on weight loss and BMI in diabetic patients.

Our study showed that the mean BMI and weight loss at both short- and long-term follow-ups were not influenced by patients' age or underlying hypertension and hyperlipidemia. However, male patients experienced more pronounced weight loss than females at the one-year follow-up. Furthermore, the mean weight loss was greater with GB (RYGB and MGB) than LSG at one-year follow-up, which is in line with findings from Tabesh et al. [20] and Soong et al. [21], who reported greater weight loss following GB compared to LSG. On the other hand, in our study, the type of MS has no effect on the mean weight loss at four- to five-year follow-up. This is consistent with the SLEEVEPASS [22] and SM-BOSS [23] clinical trials, which revealed comparable long-term weight loss (at five years) between RYGB and LSG procedures.

Despite the increasing use of various metabolic surgical procedures, the underlying mechanisms of their effectiveness have not been fully understood. However, based on our findings, GB resulted in greater weight loss compared with LSG in a short time, which may be attributed to the malabsorption effects of this procedure [20]. Since the anatomical structure of the gastrointestinal tract is modified in gastric bypass surgery, the capacity of the stomach decreases and prevents the passage of food through the duodenum and proximal jejunum, decreasing the percentage of absorption of lipids and carbohydrates, resulting in rapid, effective, and long‐term weight loss [24].

One of the main goals of MS is the improvement of obesity-related comorbidities such as T2DM, hypertension, dyslipidemia, and CVDs [20,25]. In our study, a significant improvement of FBS and HbA1c was found following MS in T2DM patients, and this reduction remained stable in the long term. Furthermore, a high rate of diabetes remission was observed one year postoperatively (75.2% remission and 24.8% improvement), which was sustained in the long term (69.0% remission and 24.1% improvement). These results were consistent with previous studies by Alamro et al. [2], Park et al. [26], and Vennapusa [18], which reported diabetes remission in 78.2%, 72%, and 90.8%, respectively, one year after MS. Our findings are in accordance with the results of previous studies, which have reported that MS represents a viable therapeutic option for T2DM and achieves sustainable glycemic control [5,20,27]. Moreover, in our study, the mean FBS decreased from 157.22 mg/dL at baseline to 99.91 mg/dL one year postoperatively, and HbA1c decreased from 7.28% to 5.46%, which are below the diagnostic criteria for T2DM as defined by ADA [13].

Evidence suggests that MS may improve glucose control and insulin sensitivity through various mechanisms, including the reduction of ghrelin, increased levels of satiety hormones such as glucagon-like peptide 1 (GLP-1) and peptide YY (PYY), limited intestinal absorption, reduced hepatic gluconeogenesis, and the elimination of glucotoxic effects on pancreatic beta cells [8,28]. Additionally, alterations in bile acid recycling and increased serum bile acid concentration [29] may contribute to the diabetes improvement. In our study, reductions in FBS and HbA1c were higher in older patients, which could be attributed to different physiological responses associated with aging. Further studies on the underlying mechanisms of diabetes improvement and glycemic control after MS will enhance our understanding of metabolic surgeries and help identify novel therapeutic targets for metabolic disorders.

Our results demonstrated a significant reduction in both SBP and DBP one year postoperatively compared to preoperative values, and this improvement remained stable during long-term follow-up for SBP. At the one-year follow-up, remission and improvement of hypertension were observed in 48.2% and 47.3% of patients, respectively. Similarly, in the study by Sun et al. [30] and Jeon et al. [31], significant improvements in BP and a reduction in the use of antihypertensive medications were observed after MS. Improvement of hypertension in diabetic patients after MS was also reported in other studies [2,32]. Since hypertension is one of the components of metabolic syndrome and is associated with obesity, and given that many studies have shown that weight loss can reduce BP [33,34], our results support the notion that MS is an effective strategy for controlling BP in patients with morbid obesity.

Our findings demonstrate that MS significantly reduced TC, LDL-C, and TG levels while increasing HDL-C in diabetic patients over a four- to five-year follow-up period. Furthermore, the remission and improvement rates for hyperlipidemia were 35.1% and 54.4%, respectively, in a one-year follow-up. In agreement with the present study, Jiménez et al. [35], Alamro et al. [2], Parmar et al. [27], and Tabesh et al. [20] reported similar and significant improvement in lipid profile parameters at short- and long-term follow-ups after MS. The improvement in the lipid profile might be attributed to the reduction in gastric volume, leading to decreased gastric lipase production, or the malabsorptive influence of MS [36].

In our study, the mean FRS at both one-year and long-term (four to five years) follow-up was significantly lower than the preoperative values. Our findings also indicated a significantly greater reduction of the FRS in males and patients with hypertension and hyperlipidemia one year following MS. Additionally, a direct and significant correlation was found between FRS changes and patient age. These results suggest that MS can help reduce the risk of CADs in T2DM, particularly in older patients and those with hypertension and hyperlipidemia, which are all known cardiovascular risk factors.

Consistent with these findings, Alamro et al. reported a significant decrease in FRS one year after MS [2]. Similarly, in a study by Schiavon et al., MS led to a notable reduction in FRS among obese patients with hypertension, with or without diabetes [37]. Aminian et al. also reported a 27% decrease in 10-year CVD risk after RYGB in diabetic obese patients [38]. Reduction of cardiovascular risk following MS has also been documented in other studies [11,12]. It has also been reported that a decrease in BP and lipid profile reduces cardiovascular risk in patients undergoing MS [28]. Therefore, this evidence altogether shows that MS is a promising and valuable strategy not only for sustainable weight loss but also for reducing FRS in patients with T2DM and morbid obesity, which indicates the risk of CVDs. However, since the CVD risk varies depending on dietary patterns, lifestyle, and ethnic-racial origin, generalizing these results to other populations remains challenging [39]. Moreover, during the one-year follow-up after MS, no progression or new onset of CVDs was observed in our patients. Although these findings suggest a positive effect of MS on cardiac function in T2DM patients, evaluating the long-term status of cardiovascular disease requires extended follow-up.

In the present study, levels of liver enzymes, including AST, ALT, and ALP, significantly decreased at the one-year follow-up after MS. The changes in AST and ALT remained stable throughout the long-term follow-up. These findings are consistent with studies by Alamro et al. [2], Kirkpatrick et al. [40], and Głuszyńska et al. [41], which demonstrated that MS improves liver function in obese diabetic patients. A reduction in serum transaminase levels may help prevent liver fibrosis and non-alcoholic fatty liver disease (NAFLD) progression [42]. These results suggest that MS is an effective treatment for hepatic manifestations of metabolic syndrome in patients with morbid obesity and diabetes. However, the underlying mechanisms of improved liver function following MS should be further investigated in future studies.

In our study, the type of MS had no effect on the remission rate of diabetes and hyperlipidemia at both the one-year and four- to five-year follow-ups. However, the remission rate of hypertension was higher in the GB group compared to the LSG group (one-year follow-up: 62.1% vs. 33.3% and long-term: 74.9% vs. 14.3%). In the study by Khalaj et al., both LSG and RYGB procedures showed similar effects on diabetes remission (52.3% and 63.8%, respectively) but different effects on hypertension remission (39.1% vs. 54.7%, respectively), two years postoperatively [25]. Another study analyzing five-year outcomes from the SLEEVEPASS and SM-BOSS trials revealed that T2DM remission rates were similar in RYGB and LSG, but the hypertension remission rate was higher in the RYGB group than in the LSG group (60.3% vs. 44.9%, respectively) [43]. Similarly, Tabesh et al. revealed that the significant improvement in FBS, lipid profile, and dyslipidemia after MS did not differ by surgical technique (LSG and GB) at one-year follow-up [20].

In our study, a low rate of surgery-related complications was observed (3.8%) during the follow-up period, and no mortality was reported, indicating that MS is a safe and effective intervention in T2DM patients. This relatively low complication rate is in contrast to the findings of Li et al. [32] and Parmar et al. [27], who reported postoperative complication rates of 11% and 15.9% in obese diabetic patients, respectively. Additionally, a large-scale study reported a five-year complication rate of 8.94% after RYGB and LSG procedures [44]. Although the risks associated with MS have significantly declined in the era of laparoscopic techniques [6] and several studies have reported no surgery-related mortality [6,27,32], postoperative complications may vary considerably depending on the type of procedure and patients' characteristics. The limited follow-up periods may have led to the underreporting of postoperative complications in our study.

Collectively, these findings confirm the long-term metabolic and cardiovascular benefits of MS in T2DM patients, with minimal complications and sustained improvement of obesity-related comorbidities. Ultimately, this multicenter follow-up study evaluated various metabolic and obesity outcomes following common MS in T2DM patients. To the best of our knowledge, this is the first comprehensive study for the evaluation of the impact of MS on FRS in diabetic patients in Iran.

This study had several limitations. First, the retrospective design prevented access to relevant data, such as specific laboratory tests (albumin, ferritin, and creatinine levels) that were not available for all patients, as well as information on dietary intake and physical activity. Additionally, missing data and incomplete follow-up led to the exclusion of several patients. Second, the findings are based on data from an MS center in Ahvaz, Iran, which may limit the generalizability of the results to other national and international populations. Third, the use of convenience sampling led to uneven subgroup distribution (e.g., by surgery type), which may have introduced bias.

Moreover, due to the limited sample size and the retrospective nature of the study, more complex analysis and subgroup exploration were not feasible. Additionally, given the observational nature of this study, there may be potential confounding factors that could have influenced the results. These factors were not controlled for, which is a limitation of the study. Future research should aim to control for confounders using appropriate statistical methods to minimize bias and improve the validity of findings. Therefore, randomized controlled trials and large-scale prospective studies are recommended to achieve more reliable and generalizable results. Future research should also focus on identifying personalized indicators for MS candidacy, rather than relying solely on high BMI and diabetes.

## Conclusions

The findings of this study demonstrate that MS in obese patients with T2DM results in significant and sustained weight loss, improved glycemic control, BP, lipid profile, and liver enzyme levels, as well as the remission and improvement of obesity-related conditions such as T2DM, hypertension, and dyslipidemia. It also results in a reduced cardiovascular risk based on the FRS. These beneficial outcomes persisted during the four- to five-year postoperative follow-up, confirming the long-term efficacy of this intervention in diabetic patients with various levels of obesity (Class I-III). These results highlight the promising role of MS in the management of obesity and its complications in the diabetic population.
